# Comparative analysis of the therapeutic effect of antibiotic bone cement on Wagner grade 3 or 4 diabetic foot ulcer in heel and non-heel areas: a retrospective cohort study

**DOI:** 10.3389/fendo.2025.1662731

**Published:** 2025-11-26

**Authors:** Yang Jian, Li Li, Wei Chen, Wenyu An, Runxue Guan, Yanji Zhang, Jiarui Wei, Shusen Chang, Jian Zhou, Kaiyu Nie, Chengliang Deng, Zairong Wei

**Affiliations:** 1 Department of Burns and Plastic Surgery, Affiliated Hospital of Zunyi Medical University, Zunyi, Guizhou, China; 2 The 2011 Collaborative Innovation Center of Tissue Damage Repair and Regeneration Medicine, Affiliated Hospital of Zunyi Medical University, Zunyi, Guizhou, China; 3 The Collaborative Innovation Center of Tissue Damage Repair and Regeneration Medicine, Zunyi Medical University, Zunyi, Guizhou, China; 4 Guizhou Biofabrication Laboratory, Affiliated Hospital of Zunyi Medical University, Zunyi, Guizhou, China

**Keywords:** diabetic foot ulcer, antibiotic bone cement, major amputation, free flap, tibial transverse transport

## Abstract

**Background:**

Heel diabetic foot ulcers (hDFU) represent a particularly severe form of DFU, characterized by prolonged healing times and a significantly elevated risk of major amputation. Effective strategies to control infection and improve outcomes in this high-risk population are urgently needed. While antibiotic bone cement (ABC) has emerged as a promising therapy for general DFU, its specific efficacy for hDFU remains unverified. This study aims to evaluate the clinical efficacy of ABC-based management for hDFU compared to non-heel DFU (nhDFU) and to identify risk factors for major amputation.

**Methods:**

We conducted a retrospective cohort study of 77 patients with severe (Wagner grade 3 or 4) DFUs treated with an ABC protocol. Patients were stratified into hDFU (n=35) and nhDFU (n=42) groups. Propensity score matching (PSM) was used to balance baseline characteristics. Outcomes included major amputation rates, length of stay (LOS), infection control [measured by white blood cell (WBC) count], and risk factors analyzed via modified Poisson regression.

**Results:**

At baseline, the hDFU group presented with greater disease severity, evidenced by significantly lower albumin levels (29.56 ± 6.45 g/L vs. 32.49 ± 5.25 g/L, *P* = 0.03) and higher WBC counts (Median: 14.86 vs. 10.32 × 10^9^/L, *P* = 0.002). After PSM, the major amputation rate was not significantly different between the hDFU and nhDFU groups (12% vs. 8%, *P* = 1.0). ABC treatment significantly reduced WBC counts in both groups (*P<* 0.01), indicating effective infection control. Multivariate analysis identified alcohol abuse as an independent risk factor for major amputation both before [RR = 1.095, 95% confidence interval (CI): 1.011-1.186] and after PSM (RR = 1.123, 95% CI: 1.017-1.240). Hypoalbuminemia was also associated with increased amputation risk.

**Conclusion:**

An ABC-based management strategy is effective for severe hDFU, demonstrating comparable major amputation rates to nhDFU despite more severe initial presentations. It facilitates infection control and may contribute to shortened hospitalization. Clinicians should address modifiable risk factors, particularly alcohol abuse and hypoalbuminemia, to further improve limb salvage outcomes.

## 1 Introduction

Diabetic foot ulcer (DFU) is a serious chronic complication of diabetes mellitus (DM),affecting approximately 19-34% of DM patients (1). As the leading cause of amputation and death among individuals with DM (2), DFU is responsible for 85% of the DM-related amputations that occur globally every 20 seconds (3, 4). The annual mortality rate for patients with DFU is as high as 11%, rising to 22% among those who undergo amputation (5). The economic burden is equally staggering; in 2017, DFU accounted for approximately one-third of the $727 billion in global DM-related medical expenditures (6). Therefore, the management of DFU presents a formidable challenge.

This challenge is particularly pronounced in heel DFU (hDFU). Evidence indicates a progressive increase in healing time from toe to heel, with median healing times of 147 days for toe ulcers, 188 days for midfoot ulcers, and 237 days for hDFU (7). This delayed healing is associated with an elevated risk of infection (8), and hDFU has been identified as a risk factor for major amputation (9). A prospective study by Saleem et al. (10) revealed that 43% of major amputations occurred in hDFU cases, underscoring its poor prognosis. The higher amputation rate is largely attributed to the greater risk of deep infections involving the ankle joint in this region (11). Consequently, achieving rapid and effective infection control is a strategic priority in preventing major amputations in hDFU.

Antibiotic bone cement (ABC) has emerged as a promising adjunct therapy in the management of DFU (12). Several clinical studies have documented its effectiveness in controlling localized infection, reducing inflammatory markers, shortening hospital length of stay (LOS), lowering the rate of major amputations, and ultimately promoting wound healing (12–14). Moreover, compared with negative pressure wound therapy (NPWT), ABC showed better clinical effects in controlling infection, promoting wound healing, reducing inflammation levels, and promoting the expression of growth factors in wounds (15, 16). The mechanism is attributed to the local and sustained release of high-concentration antibiotics, which effectively targets biofilm-associated infections (13, 17). Moreover, ABC has been proven to promote the polarization of M2 macrophages (18), increase the expression of growth factors in DFU (16, 19, 20), and promote the formation of the induced membrane (13, 18, 19), which is beneficial to the healing and repair of DFU.

The existing body of evidence for ABC is predominantly derived from studies on heterogeneous DFU populations, without a specific focus on the high-risk hDFU subgroup. This represents a significant knowledge gap. Given the anatomical and physiological particularities of the heel region—such as thinner soft tissue, poorer blood supply, and higher mechanical load—the pathophysiology and healing challenges of hDFUs are distinct. It is therefore unclear whether the promising results of ABC from general DFU studies can be directly extrapolated to hDFUs. A direct, comparative investigation of ABC’s efficacy in hDFU versus non-heel DFU (nhDFU) is lacking but is critically needed to optimize treatment strategies for this vulnerable population.

In this study, we implemented a wound surgical integrated treatment (WSIT) protocol incorporating ABC for DFU management (18, 21). To address the identified research gap, this study specifically aims to evaluate and compare the efficacy of ABC in the treatment of hDFU and nhDFU, and to identify the risk factors for major amputation. Our work provides a novel, targeted evaluation of a promising therapy in the DFU subgroup that stands to benefit the most, thereby aiming to refine clinical practice and improve outcomes for these high-risk patients.

## 2 Patients and methods

### 2.1 Ethics and registration statement

The study was approved by the Ethics Committees of the Affiliated Hospital of Zunyi Medical University (KLL-2024-694). This study was conducted in accordance with the principles of the Declaration of Helsinki. The need for informed consent of the participants was waived due to the retrospective nature of the research. This work has been reported in line with the Strengthening the Reporting of Cohort, Cross-sectional and Case - control Studies in Surgery (STROCSS) criteria (22).

### 2.2 Study design and patient

**Figure 1:**
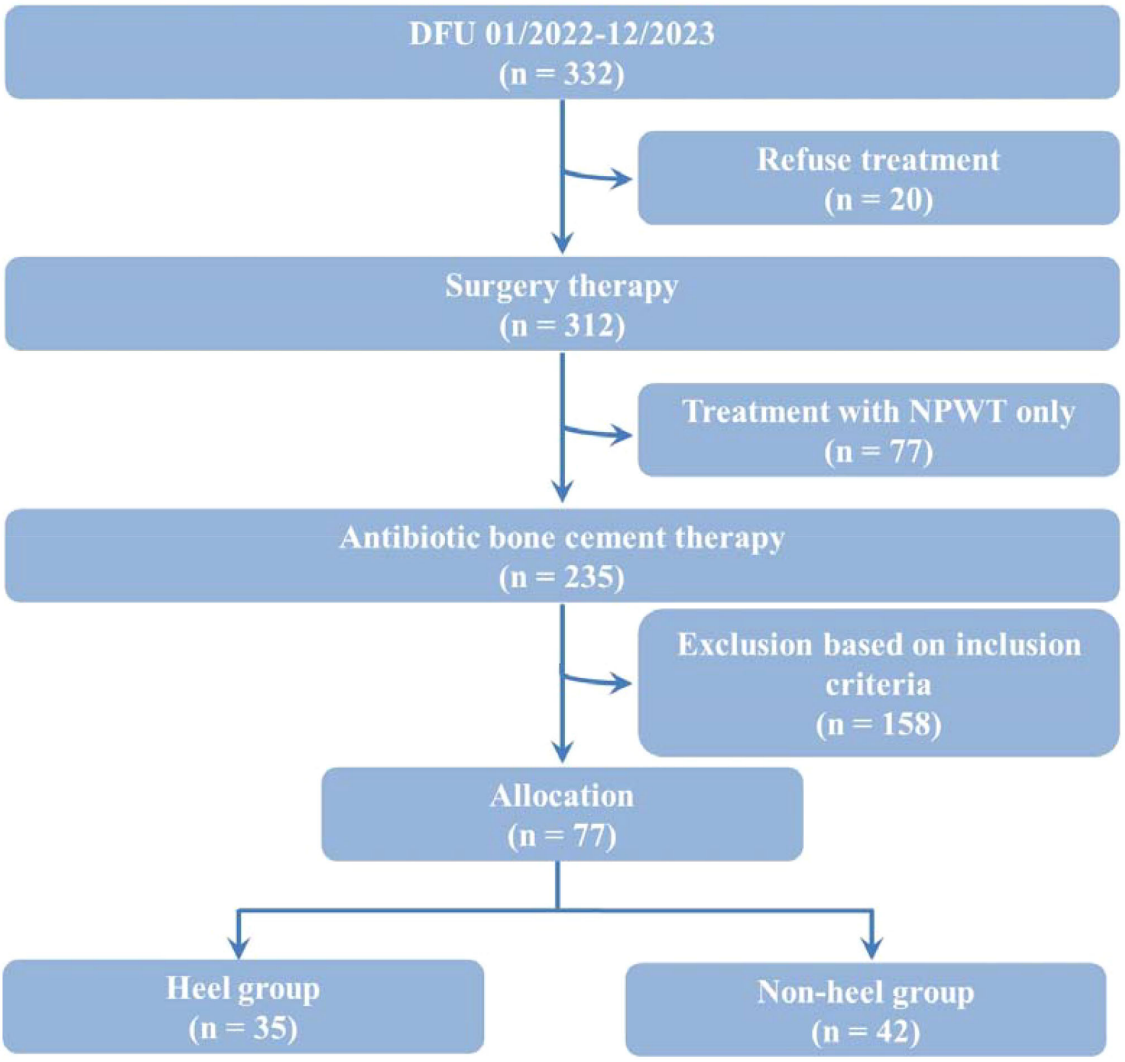
Schematic flow diagram of the study. DFU, diabetic foot ulcer; NPWT, negative pressure wound therapy.

This retrospective cohort study initially screened 332 patients with DFU between January 2022 and December 2023. Of these, 255 were excluded for the following reasons: 20 (6.0%) declined treatment, 77 (23.2%) received NPWT only, and 158 were from the ABC-treated group but did not meet the inclusion criteria. Consequently, the final study population comprised 77 patients (23.2% of the initial cohort) who received ABC treatment as part of the WSIT protocol. These participants were stratified into a hDFU group (n = 35) and a nhDFU group (n = 42) based on ulcer location (**Figure 1**).

To ensure a homogeneous study population for assessing the efficacy of ABC, we applied the following inclusion and exclusion criteria: Inclusion criteria: (1) Age between 18 and 80 years; (2) male or female; (3) diagnosed with type 1 or type 2 DM; (4) DFU classified as Wagner grade 3 or 4; (5) Underwent at least one application of ABC following surgical debridement. Exclusion criteria: (1) Incomplete clinical data that precluded outcome assessment (e.g., missing key laboratory results or loss to follow-up before wound repair); (2) Received non-surgical conservative management only; (3) Presence of active malignant tumors; (4) Admission to the intensive care unit (ICU) for reasons unrelated to the primary DFU management (e.g., septic shock from another source, acute cardiac events), to avoid confounding the length of stay (LOS) analysis.

### 2.3 Data collection and outcomes

Data for all eligible patients were retrospectively extracted from the hospital’s electronic medical record system. To ensure consistency in data acquisition, all laboratory measurements (e.g., hemoglobin, albumin, white blood cell count) were performed using standardized, hospital-calibrated equipment following the institution’s routine clinical protocols. The following information was collected: (1) characteristics at admission, including age (years), gender, DM duration (years), DFU duration (months), ankle brachial index (ABI), smoking, alcohol abuse, ulcer site, Wagner grade, and diabetic nephropathy; (2) ulcer size (cm2) after the first debridement; (3) laboratory examination, including hemoglobin (Hb) (g/L), albumin (g/L), fasting blood glucose (FBG) (mmol/L), glycosylated hemoglobin A1c (HbA1c) (%), and wound bacterial culture; (4) clinical outcome, including major amputation, wound repair modalities, LOS (days), and changes in white blood cell (WBC) levels (× 10^9^/L) before and after ABC treatment.

The primary outcome was the incidence of major amputation during the perioperative period of definitive wound management. This period was defined as the interval from the patient’s first admission for the index DFU to final discharge following definitive surgical repair (e.g., free flap, skin graft, or amputation). A major amputation was defined in accordance with the International Working Group on the Diabetic Foot guidelines (2023) as any resection proximal to the ankle (23).

Secondary outcomes included wound repair modalities for DFU, WBC changes, and LOS. The DFU repair modalities included skin grafting, free flap, and suture alone or in combination with tibial transverse transport (TTT) or nerve decompressive surgery of the lower limbs (NDSLL) or adipose-derived stromal vascular fraction (aSVF). Changes in WBC were assessed by calculating the difference between the WBC levels measured during the first admission and those recorded during the second admission. For patients with a single hospitalization, WBC levels obtained on postoperative day 3 following ABC application were used as a surrogate for second-admission WBC measurements. Total LOS was defined as the cumulative duration encompassing all DFU-related hospitalizations from initial admission until discharge following definitive ulcer repair. These data were collected from the electronic medical record system.

### 2.4 Surgical techniques

**Figure 2:**
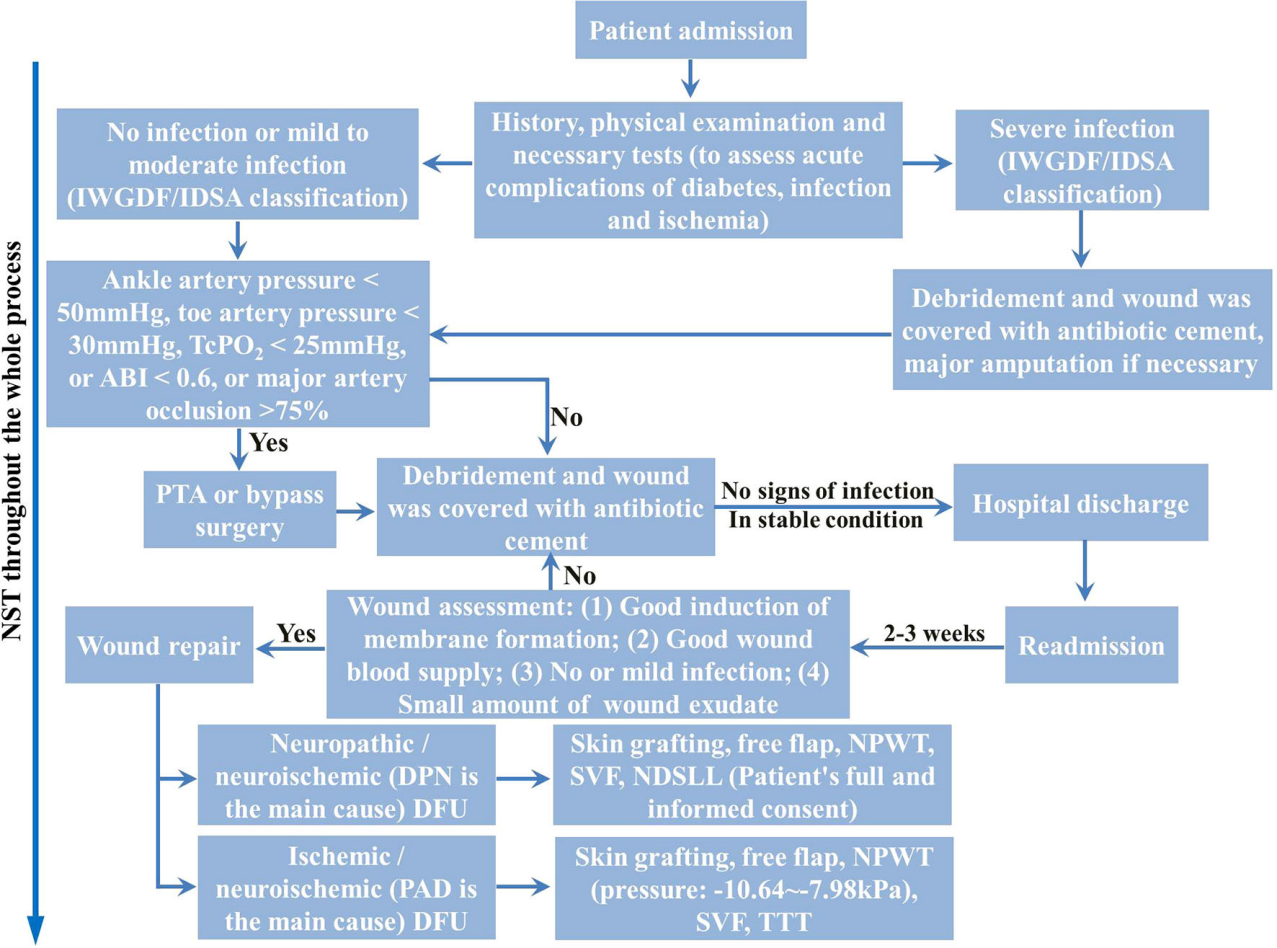
The ABC-based WSIT for DFU management. WSIT, wound surgical integrated treatment; IWGDF/IDSA, International Working Group on the Diabetic Foot/Infectious Diseases Society of America; TcPO_2_, transcutaneous oxygen pressure; ABI, ankle brachial index; PTA, percutaneous transluminal angioplasty; DPN, diabetic peripheral neuropathy; NPWT, negative pressure wound therapy; SVF, stromal vascular fraction; NDSLL, nerve decompressive surgery of the lower limbs; PAD, peripheral arterial disease; TTT, tibial transverse transport; NST, nutrition support therapy.

All surgical procedures were performed by a dedicated multidisciplinary WSIT team following a standardized institutional protocol (**Figure 2**) (18, 21). Briefly, a thorough history, physical examination, and laboratory investigations were conducted to promptly assess acute diabetic complications, infection, and ischemia. Subsequently, a multidisciplinary team meeting was convened to assess the patient’s overall clinical status, formulate a DFU-tailored surgical plan, and determine the optimal timing and approach for surgery.

For patients with severe foot infections, immediate radical debridement was performed. In cases of critical limb ischemia with less severe infection, revascularization via percutaneous transluminal angioplasty (PTA) or bypass surgery preceded definitive debridement. Indications for revascularization included (18): ankle artery pressure< 50 mmHg, toe artery pressure< 30 mmHg, transcutaneous oxygen pressure< 25 mmHg, ABI< 0.6, or >75% luminal occlusion of major arteries. Following debridement in all patients, ABC was applied to fill and cover the DFU. The outer dressings were removed 3–5 days postoperatively for wound assessment. Patients showing no complications were discharged and scheduled for follow-up and secondary wound repair 2–3 weeks later. If severe wound exudation, foul odor, or other signs of infection were observed during outpatient care, repeat debridement was performed. NPWT was commonly used for wound bed preparation before secondary repair, with caution exercised in cases of ischemic DFU (24).

The specific approach for Phase II definitive wound repair was determined by the wound characteristics and patient factors. Free flap reconstruction was employed for wounds with exposed nerves, bone, or tendons, provided that the lower limb vascular peak flow rate exceeded 40 cm/s (18). For wounds without such exposure and with a well-vascularized wound bed, skin grafting was utilized, or primary suture was applied for small defects. TTT was considered for selected cases with ischemia (21, 25). In patients presenting with diabetic peripheral neuropathy, a positive Tinel sign, and a history of ineffective conservative management, NDSLL was offered based on patient consent (25). Subsequently, aSVF therapy could be administered to address any residual wound areas (26). All these procedures were performed by the integrated WSIT team, obviating the need for external consultations (18). Comprehensive nutritional support was maintained throughout the entire treatment period.

### 2.5 Statistical analysis

All statistical analyses were performed using SPSS Statistics version 29.0 (IBM Corp., Armonk, NY, USA) and R software version 4.4.2 (R Foundation for Statistical Computing, Vienna, Austria). A two-sided P-value< 0.05 was considered statistically significant for all tests.

Continuous variables were tested for normality using the Shapiro-Wilk test. Normally distributed data are presented as mean ± standard deviation (SD) and were compared between the two groups using the Independent Samples Student’s *t*-test. Non-normally distributed data are presented as median and interquartile range (IQR) and were compared using the Mann-Whitney *U* test. Categorical variables are presented as counts and percentages (n, %) and were compared using the Pearson Chi-square test or Fisher’s exact test, as appropriate.

To minimize selection bias and balance baseline characteristics between the hDFU and nhDFU groups, 1:1 propensity score matching (PSM) was performed using the MatchIt package in R. The matching was conducted using the nearest neighbor method with a caliper width of 0.2 of the standard deviation of the logit of the propensity score (27). Balance between the matched groups was assessed using the standardized mean difference (SMD), where an SMD< 0.1 was considered indicative of good balance (28).

To identify risk factors for major amputation, we used modified Poisson regression with robust error variance to directly calculate relative risks (RR) and 95% confidence intervals (CI). All variables with a *P*-value< 0.10 in univariate analysis, or those with an SMD > 0.2 after PSM indicating potential residual imbalance, were included in the multivariable modified Poisson regression model. The final model was built using a backward stepwise selection process to retain variables significantly associated with the outcome.

## 3 Results

### 3.1 Patient characteristics

The study enrolled 77 patients with DFU. The cohort had a mean age of 57.83 ± 9.91 years, was predominantly male (70.1%), and consisted entirely of individuals with type 2 diabetes. Key baseline characteristics, including diabetes duration, ulcer size, and laboratory values, are summarized in **Table 1**. The majority of ulcers were Wagner grade 4 (55.8%), and wound bacterial cultures most frequently revealed mixed infections (28.6%), followed by *Staphylococcus aureus* (24.7%).

**Table 1:** Clinical characteristics of the enrolled patients.

Characteristic	Heel group (n=35)	Non-heel group (n=42)	*P*-value
Age (years) ( $\bar{x}$ ± *s*)	57.09 ± 8.98	58.45 ± 10.69	0.55 ^a^
Gender, n (%)			0.47 ^b^
Male	26 (74.29)	28 (66.67)	
Female	9 (25.71)	14 (33.33)	
DM duration (years) [*M*(*Q_1_*,*Q_3_*)]	9 (6.00, 10.00)	10 (4.75, 10.33)	0.82 ^c^
DFU duration (months) [*M*(*Q_1_*,*Q_3_*)]	1 (0.33, 2.00)	0.75 (0.38, 2.00)	0.89 ^c^
ABI ( $\bar{x}$ ± *s*)	0.78 ± 0.14	0.81 ± 0.13	0.41 ^a^
Ulcer size (cm^2^) ( $\bar{x}$ ± *s*)	62.89 ± 33	72.6 ± 31.03	0.19 ^a^
Hb (g/L) ( $\bar{x}$ ± *s*)	99.2 ± 23.94	105.62 ± 19.36	0.20 ^a^
Albumin (g/L)( $\bar{x}$ ± *s*)	29.56 ± 6.45	32.49 ± 5.25	0.03 ^a^
Admission FBG (mmol/L) ( $\bar{x}$ ± *s*)	11.56 ± 3.83	11.15 ± 4.51	0.67 ^a^
HbA1c (%) [*M*(*Q_1_*,*Q_3_*)]	8.2 (7.50, 10.60)	8.35 (7.48, 9.70)	0.70 ^c^
First admission WBC (×10^9^/L) [*M*(*Q_1_*,*Q_3_*)]	14.86 (9.58, 18.23)	10.32 (7.95, 14.57)	0.014 ^c^
Smoking, n (%)			0.82 ^b^
Smoker	16 (45.71)	18 (42.86)	
Non-smoker	19 (54.29)	24 (57.14)	
Alcohol abuse, n (%)			0.81 ^b^
Drinker	14 (40.00)	15 (35.71)	
Non-drinker	21 (60.00)	27 (64.29)	
Ulcer site, n (%)			
Left	19 (54.29)	22 (52.38)	1.00 ^b^
Right	16 (45.71)	20 (47.62)	
Wagner grade, n (%)			
3grade	13 (37.14)	21 (50.00)	0.36 ^b^
4grade	22 (62.86)	21 (20.00)	
Diabetic nephropathy, n (%)	6 (17.14)	5 (11.90)	0.75 ^b^
BC, n (%)			0.97 ^d^
Negative	4 (11.43)	6 (14.29)	
SA	9 (25.71)	10 (23.81)	
PA	2 (5.71)	4 (9.52)	
* Baumanii*	3 (8.57)	4 (9.52)	
EC	2 (5.71)	3 (7.14)	
MI	12 (34.29)	10 (23.81)	
Other	3 (8.57)	5 (11.90)	

DM, diabetes mellitus; DFU, diabetic foot ulcer; ABI, ankle-brachial index; Hb, hemoglobin; FBG, fasting blood glucose; HbA1c, glycosylated hemoglobin A1c; WBC, white blood cell; BC, bacterial culture; SA, *Staphylococcus aureus*; PA, *Pseudomonas aeruginosa*; EC, *Escherichia coli*; MI, mixed infection. ^a^ the student’s *t*-test was used to compare heel group to non-heel group; ^b^ the Pearson chi-square test was used for comparison between the two groups; ^c^ the Mann-Whitney *U* test was used for comparison between the two groups; ^d^ Fisher’s exact test was used for comparison between the two groups.

While most baseline characteristics were comparable between the hDFU and nhDFU groups, significant differences were observed in two key metrics at admission. The hDFU group presented with significantly lower albumin levels (29.56 ± 6.45 g/L vs. 32.49 ± 5.25 g/L; *P* = 0.03) and higher WBC counts [median: 14.86 (IQR: 9.58-18.23) × 10^9^/L vs. 10.32 (IQR: 7.95-14.57) × 10^9^/L; *P* = 0.002]. To account for these baseline imbalances, a 1:1 PSM was performed, which yielded 25 well-matched pairs in each group (total n=50). The baseline characteristics of this matched cohort were balanced, with no significant differences remaining (**Supplementary Table S1**).

### 3.2 Clinical outcomes

**Figure 3:**
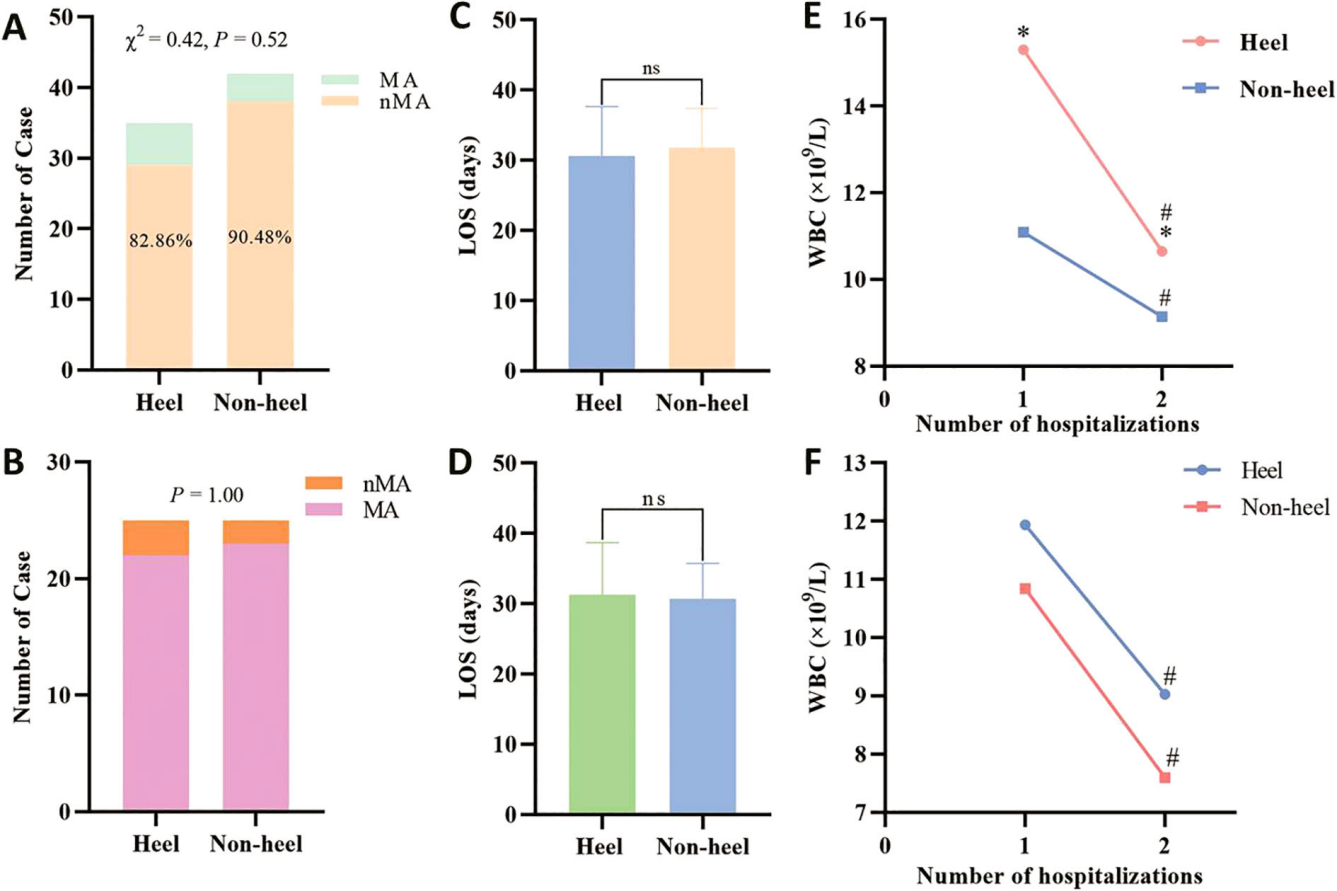
Comparison of treatment outcomes. **(A)** Comparison of major amputation rates before PSM, and the chi-square test was used; **(B)** Comparison of major amputation rates after PSM, and Fisher’s exact test was used; **(C)** Comparison of LOS before PSM; **(D)** Comparison of LOS after PSM, and the student’s *t*-test was used; **(E)** Comparison of WBC before PSM, and the student’s *t*-test was used. * There was a significant difference in the comparison between the two groups; # There was a significant difference between the preoperative and postoperative comparisons within the group; **(F)** Comparison of WBC before PSM, and the student’s *t*-test was used. # There was a significant difference between the preoperative and postoperative comparisons within the group. MA, major amputation; nMA, none major amputation; LOS, length of stay; WBC, white blood cells.

The incidence of major amputation was not significantly different between the hDFU and nhDFU groups, either before (17.14% vs. 9.52%; *P* = 0.52) or after PSM (12.00% vs. 8.00%; *P* = 1.00) (**Table 2**, **Figures 3A, B**). The distribution of wound repair modalities across the entire cohort is detailed in **Table 3**. The most common procedures were skin grafting (31.16%) and free flap repair (28.57%). There was no significant difference in the modality distribution between groups before or after PSM (*P* = 0.47 and *P* = 0.24, respectively). No significant differences were found in the total LOS between groups before (30.66 ± 6.99 vs. 31.86 ± 5.55 days; *P* = 0.40) or after PSM (31.32 ± 7.35 vs. 30.72 ± 5.01 days; *P* = 0.74) (**Figures 3C, D**). ABC treatment significantly reduced WBC counts in both groups (**Figures 3E, F**). Before PSM, WBC levels decreased from 14.86 (9.58-18.23) to 10.34 (7.40-14.17) × 10^9^/L in the hDFU group (*P* = 0.008) and from 10.32 (7.95-14.57) to 7.51 (6.07-9.71) × 10^9^/L in the nhDFU group (*P* = 0.0003). Notably, both pre- and post-treatment WBC levels were significantly higher in the hDFU group (*P* = 0.014 and *P =* 0.002, respectively). After PSM, a significant WBC reduction was maintained in both the hDFU (11.94 ± 3.91 to 9.03 ± 2.80 × 10^9^/L; *P* = 0.004) and nhDFU groups (10.84 ± 4.32 to 7.60 ± 2.83 × 10^9^/L; *P* = 0.003). The magnitude of reduction between the matched groups was not significantly different (**Figure 3F**).

**Table 2:** Differences in major amputation rates between groups.

Outcome	Before PSM		RR (95% CI)	*P*-value	After PSM		RR (95% CI)	*P*-value
	Heel group (n = 35)	Non-heel group (n = 42)			Heel group (n = 25)	Non-heel group (n = 25)		
MA	6 (17.14)	4 (9.52)	1.8 (0.55, 5.87)	0.52^a^	3 (12.00)	2 (8.00)	1.5 (0.27,8.21)	1.00^b^
nMA	29 (82.86)	38 (90.48)			22 (88.00)	23 (92.00)		

a Yates continuity correction was used for comparison between the two groups.

b Fisher’s exact test was used for comparison between the two groups.

**Table 3:** Comparison of treatment outcomes.

Outcome	Before PSM		*P*-value	After PSM		*P*-value
	Heel group (n = 35)	Non-heel group (n = 42)		Heel group (n = 25)	Non-heel group (n = 25)	
Major amputation	6 (17.14)	4 (9.52)	0.47*	3 (12.00)	2 (8.00)	0.24*
Free flap	12 (34.29)	10 (23.81)		9 (36.00)	9 (36.00)	
Skin grafting	11 (31.43)	13 (30.95)		8 (32.00)	5 (20.00)	
Suture	1 (2.86)	5 (11.90)		1 (4.00)	4 (16.00)	
TTT	1 (2.86)	5 (11.90)		1 (4.00)	3 (12.00)	
Combined NDSLL	3 (8.57)	3 (7.14)		3 (12.00)	0	
Combined SVF	1 (2.86)	2 (4.76)		0	2 (8.00)	

PSM, propensity score matching; TTT, tibial transverse transport; NDSLL, nerve decompressive surgery of the lower limbs; SVF, stromal vascular fraction gel.

*Fisher’s exact test was used for comparison between the two groups.

### 3.3 Risk factors for major amputations

The associations between potential risk factors and major amputation are detailed in **Table 4**. In the univariate analysis, hypoalbuminemia was significantly associated with major amputation both before (RR = 1.008, 95% CI: 1.001-1.017; *P* = 0.037) and after PSM (RR = 1.010, 95% CI: 1.000-1.021; *P* = 0.047). Alcohol abuse was also identified as a risk factor in the univariate model after PSM (RR = 1.077, 95% CI: 1.012-1.146; *P* = 0.020). In the multivariable analysis, alcohol abuse remained an independent risk factor for major amputation, both before (RR = 1.095, 95% CI: 1.011-1.186; P = 0.026) and after PSM (RR = 1.123, 95% CI: 1.017-1.240; *P* = 0.021). In contrast, mixed infection was identified as a significant factor in the multivariable model before PSM (RR = 0.873, 95% CI: 0.788-0.967; *P* = 0.009), but this association was not statistically significant in the post-matching analysis.

**Table 4:** Risk factors for major amputations.

Variable	Before PSM [relative risk (95% CI)]		After PSM [relative risk (95% CI)]	
	Univariate model	Multivariable model	Univariate model	Multivariable model
Age	1.00 (0.996, 1.003)	–	0.999 (0.997, 1.002)	–
Gender				
Male	1.034 (0.940, 1.138)	–	1.026 (0.924, 1.138)	–
Female	1 [Reference]	–	1 [Reference]	–
DM duration	0.998 (0.991, 1.004)	–	0.999 (0.995, 1.003)	–
DFU duration	1.003 (0.994, 1.012)	1.006 (0.995, 1.018)	1.005 (0.998, 1.012)	1.003 (0.991, 1.015)
ABI	1.225 (0.929, 1.614)	–	1.134 (0.89, 1.446)	–
Ulcer size	1 (0.998, 1.001)	–	1 (0.998, 1.001)	–
Hb (g/L)	1.002 (1, 1.004)	–	1.002 (0.999, 1.004)	–
Albumin	1.008 (1.001, 1.017) *	1 (0.999, 1.002)	1.01 (1, 1.021) *	1.001 (0.999, 1.002)
Admission FBG	0.997 (0.987, 1.007)	0.993(0.983, 1.003)	0.999 (0.986, 1.011)	0.997 (0.987, 1.008)
HbA1c	0.996 (0.974, 1.019)	1.002 (0.979, 1.025)	0.993 (0.967, 1.018)	0.995 (0.964, 1.027)
WBC	0.996 (0.989, 1.003)	1 (0.993, 1.007)	0.990 (0.978, 1.003)	0.994 (0.984, 1.005)
Smoking				
Smoker	1.012 (0.934, 1.096)	–	0.996 (0.909, 1.09)	–
Non-smoker	1 [Reference]	–	1 [Reference]	–
Alcohol abuse				
Drinker	1.053 (0.977, 1.135)	1.095 (1.011, 1.186)*	1.077 (1.012, 1.146) *	1.123 (1.017, 1.240)*
Non-drinker	1 [Reference]	1 [Reference]	1 [Reference]	1 [Reference]
Ulcer site				
Left	1 [Reference]	1 [Reference]	1 [Reference]	1 [Reference]
Right	1.019 (0.941, 1.104)	0.996 (0.926, 1.07)	1.045 (0.963, 1.133)	1.027 (0.934, 1.129)
Wagner grade				
3 grade	1 [Reference]	–	1 [Reference]	–
4 grade	0.934 (0.866, 1.008)	–	0.947 (0.87, 1.029)	–
DN				
Yes	1 [Reference]	1 [Reference]	1 [Reference]	1 [Reference]
Non	1.096 (0.937, 1.283)	1.141 (0.962, 1.352)	1.126 (0.923, 1.374)	1.145 (0.951, 1.379)
BC				
Negative	1 [Reference]	1 [Reference]	1 [Reference]	1 [Reference]
SA	0.974 (0.925, 1.025)	0.975 (0.912, 1.042)	1 (1, 1)	1.046 (0.918, 1.191)
PA	1 (1, 1)	1.015 (0.957, 1.076)	1 (1, 1)	1.035 (0.970, 1.104)
*Baumanii*	0.929 (0.808, 1.068)	0.925 (0.803, 1.064)	0.9 (0.741, 1.094)	0.928 (0.783, 1.101)
EC	0.9 (0.741, 1.094)	0.884 (0.722, 1.082)	1 (1, 1)	0.959 (0.835, 1.101)
MI	0.864 (0.775, 0.962)	0.873 (0.788, 0.967)*	0.893 (0.792, 1.007)	0.923 (0.821, 1.038)
Other	0.938 (0.83, 1.059)	1.027 (0.894, 1.179)	0.9 (0.741, 1.094)	1.016 (0.851, 1.213)

PSM, propensity score matching; DM, diabetes mellitus; DFU, diabetic foot ulcer; ABI, ankle-brachial index; Hb, hemoglobin; FBG, fasting blood glucose; HbA1c, glycosylated hemoglobin A1c; WBC, white blood cell; BC, bacterial culture. SA, *Staphylococcus aureus*; PA, *Pseudomonas aeruginosa*; EC, *Escherichia coli*; MI, mixed infection. * indicates *P*< 0.05.

## 4 Discussion

The management of hDFU remains a clinical challenge. Our findings, which show a persistent, though statistically non-significant, trend towards higher amputation rates in hDFU patients (17.14% vs. 9.52%) align with this consensus (9, 29). This trend mirrors the results of Saleem et al., who reported a significantly higher major amputation rate in hDFU (21.43%) compared to nhDFU (8.60%) over a 4.3-month follow-up (10). A key distinction, however, lies in the therapeutic context. While Saleem et al.’s cohort did not analyze specific treatments or Wagner grades, our study population uniformly received a standardized ABC-based WSIT protocol for severe (Wagner 3/4) ulcers. The attenuation of the amputation risk to non-significance in our cohort, despite the high-risk profile of hDFU, strongly suggests that the structured, multi-modal WSIT approach may effectively mitigate the inherent disadvantage associated with the heel location. This is further supported by comparisons with other multidisciplinary team (MDT) studies (10, 30–32). Goudie et al. and Meloni et al. reported major amputation rates of 19.05% and 20%, respectively, in hDFU cohorts (30, 33). Our observed rate of 17.14% (12.00% after PSM) in a cohort exclusively comprising Wagner 3/4 ulcers compares favorably, indicating the potential superior efficacy of integrating ABC as a core component of the MDT strategy for the most severe cases.

The potent anti-infective effect of ABC represents a plausible mechanism for its beneficial role, particularly in hDFU. Our data demonstrated a significant reduction in WBC counts following ABC application in both groups (**Figures 3E, F**), indicating its effectiveness in infection control and inflammation reduction. The biological rationale for this is multifaceted and supported by recent evidence. First, the local, sustained release of high-concentration antibiotics is a proven strategy to directly target and disrupt bacterial biofilms, which are a major barrier to healing in chronic wounds like DFU (12). This is critically important as contemporary research continues to underscore the central role of biofilms in DFU persistence and antibiotic resistance (34, 35). Second, beyond its antimicrobial action, emerging evidence suggests ABC may actively modulate the wound microenvironment. Recent studies have shown that ABC can promote the polarization of macrophages towards the M2 phenotype, which is associated with tissue repair and resolution of inflammation, and enhance the expression of growth factors crucial for healing (16, 18). We hypothesize that in hDFU—where thinner soft tissue and poorer blood supply compromise systemic antibiotic delivery and natural immune responses—this localized, multi-mechanistic action of ABC (combating biofilms and orchestrating healing) is especially critical. By providing a potent, on-site anti-biofilm and pro-healing stimulus, ABC may help to level the playing field between hDFU and nhDFU, explaining the narrowed outcome gap in our study.

The ABC-based WSIT protocol was associated with a relatively short and comparable LOS for both hDFU and nhDFU groups (**Figures 3C, D**). The lack of a significant difference in LOS suggests that the complexity of hDFU did not translate to longer hospitalization within this protocol. The mean LOS in our cohort (approximately 31 days) compares favorably with durations reported in other studies of ABC therapy, which have documented median LOS of 36 days (36) or longer healing times with conventional treatments (14, 37). Moreover, in our cohort, patients with hDFU had lower albumin levels (29.56 ± 6.45 g/L vs. 32.49 ± 5.25 g/L, *P* = 0.03) and higher median WBC levels at admission (14.86 × 10^9^/L vs. 10.32 × 10^9^/L, *P* = 0.014) compared to nhDFU patient. These suggested that the integrated WSIT approach may enhance overall treatment efficiency.

The identification of alcohol abuse as an independent risk factor for major amputation underscores a critical behavioral component in DFU management (**Table 4**). Alcohol abuse can exacerbate neuropathy, suppress immune function, and lead to poor treatment adherence (38–40). Due to reduced sensation, patients often delay treatment, allowing the infection to spread. Combined with other risk factors, this increases the likelihood of major amputation. Therefore, our findings strongly suggest that routine screening for alcohol use disorder should be implemented in patients with Wagner grade 3–4 DFUs. For those identified, integrated interventions—including brief counseling, referral to addiction specialists, and enhanced support for treatment adherence— should be considered a standard part of the multidisciplinary care plan to break the cycle and reduce amputation risk.

Our results reinforce hypoalbuminemia as a pivotal biomarker of physiological reserve and nutritional status. The lower albumin levels observed in the hDFU group, coupled with its identification as a risk factor, highlight that malnutrition and systemic inflammation are key drivers of poor outcomes (**Table 4**). Adam et al. (41) analyzed the risk factors for DFU infections extending to the leg and found that infections involving the calf were closely associated with heel ulcers and Wagner grades 3-5. Moreover, numerous factors such as ulcer size (diameter >5cm), low albumin, anemia, high HbA1c (>7.5%), high WBC levels, and ischemia have been confirmed to be associated with major amputations in DFU (29, 42, 43). Therefore, our data strongly advocate for the proactive and early involvement of a clinical nutrition team to implement personalized, protein-rich nutritional support, aiming to correct this deficit and create a metabolically favorable environment for healing.

No significant differences were found in treatment outcomes between the two groups. Interestingly, free flap repair for DFU was more common in hDFU than in nhDFU (34.29% vs. 23.81%). Free flap reconstruction for DFU is considered a viable limb-salvage strategy with a success rate of approximately 92% (44, 45). Studies have shown that free flap repair of DFU can reduce amputation and mortality rates (44–46). In our cohort, despite ABI indicating ischemia, free flap repair was still feasible after revascularization. Suh et al. (47) demonstrated that using a recanalized artery after PTA as the recipient artery for free flap repair is safe for partially occluded arteries. Even in arteries with complete occlusion, using a recanalized artery after PTA as the recipient artery still provides a 76% chance of limb preservation. Moreover, TTT is believed to improve blood circulation in the affected limb, promote wound healing in diabetic foot, and reduce the amputation rate (21, 48). In this study, only six patients underwent TTT, so its therapeutic effects cannot be measured. Nonetheless, our previous studies have shown the potential of TTT (21, 25). The application of NDSLL in DFU is controversial (49). NDSLL can improve patients’ foot pain and sensation (25), but due to the lack of guideline recommendations, informed consent from patients is essential. In addition, aSVF therapy was performed to treat residual wounds after repair. Previous study reported the effects of SVF therapy in 20 patients with chronic wounds, yielding an average healing time of 28.3 ± 9.7 days. No wound recurrence was observed over the 2–6 years of follow-up (26). In this study, 3 patients underwent SVF therapy and healed successfully, further indicating its therapeutic potential.

While our study aligns with the literature in observing a higher numerical incidence of major amputations in hDFU patients, the lack of statistical significance both before and after PSM warrants a critical appraisal of potential confounding. First, the standardized ABC-based WSIT protocol applied to all patients in this cohort might have mitigated the inherent risk disparity between hDFU and nhDFU by ensuring consistent, high-quality management. This protocol-driven approach could have particularly benefited the higher-risk hDFU group, thereby narrowing the outcome gap. Second, despite PSM balancing measured covariates, unmeasured or residual confounding might persist. For instance, the observed higher rate of free flap reconstruction in the hDFU group (34.29% vs. 23.81%) suggests that clinicians might have intuitively allocated more aggressive limb-salvage efforts to these complex cases. This differential treatment intensity, not fully captured in our propensity model, could act as a negative confounder, partially offsetting the baseline risk associated with hDFU and attenuating the observed association with amputation. Finally, although PSM improves comparability, the effective sample size post-matching limits statistical power to detect a potentially real but modest difference in amputation rates. Therefore, the non-significant p-value should not be interpreted as evidence of no difference, but rather that any existing difference was not detectable within the context of our standardized management protocol and study design.

While this study provides valuable insights into the efficacy of ABC in managing hDFU, several limitations should be acknowledged: First, the retrospective single-center design inherently carries risks of selection and information bias. Although we employed propensity score matching to balance measured confounders between the hDFU and nhDFU groups, the possibility of residual confounding due to unmeasured variables (e.g., subtle differences in soft tissue quality, biomechanical load, or patient compliance) cannot be ruled out. Second, the relatively small sample size, particularly after PSM, may have limited the statistical power of our analysis. This increases the risk of Type II errors, potentially causing us to overlook statistically significant differences in secondary outcomes or in subgroup analyses. The small sample size also constrained our ability to include a larger number of variables in the multivariable regression model for risk factors, which could lead to model overfitting. Third, the constraints of using retrospective clinical data meant that several potentially relevant parameters were not available for analysis. As such, we could not incorporate data on wound bacterial culture results, detailed revascularization outcomes (beyond the basic indication), or specific complications related to the various wound repair modalities (e.g., flap survival rates, graft failure). The use of WBC count as a primary infection marker is a further limitation, as more sensitive inflammatory markers like CRP or procalcitonin were not consistently available in the records. Finally, this study was designed to evaluate in-hospital and short-term treatment outcomes. The absence of post-discharge follow-up data prevents any assessment of long-term critical outcomes, such as ulcer recurrence rates, long-term limb salvage, functional status of the patients, or the long-term need for re-intervention. Future prospective studies with larger cohorts and long-term follow-up are warranted to validate our findings and explore these important aspects.

In conclusion, ABC-based therapy is an effective strategy for severe DFU, demonstrating comparable efficacy for hDFU and nhDFU. It facilitates infection control and reduction of systemic inflammation, leading to lower major amputation rates and a shorter length of hospital stay. This study identified alcohol abuse and hypoalbuminemia as independent risk factors for amputation. Consequently, management should include targeted interventions to address alcohol abuse and aggressive nutritional support to optimize serum albumin levels upon admission and throughout treatment. Future large-scale, multicenter prospective studies with long-term follow-up are warranted to validate these findings and evaluate critical long-term outcomes such as ulcer recurrence and patient survival.

## Data Availability

The original contributions presented in the study are included in the article/**Supplementary Material**. Further inquiries can be directed to the corresponding authors.

## References

[1] Armstrong DG Boulton AJM Bus SA . Diabetic foot ulcers and their recurrence. N Engl J Med. (2017) 376:2367–75. doi: 10.1056/NEJMra1615439, PMID: 28614678

[2] Alavi A Sibbald RG Mayer D Goodman L Botros M Armstrong DG . Diabetic foot ulcers: Part II. Management. J Am Acad Dermatol. (2014) 70:21–4. doi: 10.1016/j.jaad.2013.07.048, PMID: 24355276

[3] Rice JB Desai U Cummings AK Birnbaum HG Skornicki M Parsons NB . Burden of diabetic foot ulcers for medicare and private insurers. Diabetes Care. (2014) 37:651–8. doi: 10.2337/dc13-2176, PMID: 24186882

[4] Bakker K Apelqvist J Lipsky BA Van Netten JJ . The 2015 IWGDF guidance documents on prevention and management of foot problems in diabetes: development of an evidence-based global consensus. Diabetes Metab Res Rev. (2016) 32 Suppl 1:2–6. doi: 10.1002/dmrr.2694, PMID: 26409930

[5] Margolis DJ Malay DS Hoffstad OJ Leonard CE Macurdy T De Nava KL . Incidence of diabetic foot ulcer and lower extremity amputation among Medicare beneficiaries 2006 to 2008. In: Data Points Publication Series. Agency for Healthcare Research and Quality (US, Rockville (MD (2011). 22049565

[6] Federation, I.D . IDF Diabetes Atlas, 8th (2017). Brussels. Available online at: http://www.diabetesatlas.org [2019-01-02].

[7] Pickwell KM Siersma VD Kars M Holstein PE Schaper NC . Diabetic foot disease: impact of ulcer location on ulcer healing. Diabetes Metab Res Rev. (2013) 29:377–83. doi: 10.1002/dmrr.2400, PMID: 23390115

[8] Jeffcoate W Boyko EJ Game F Cowled P Senneville E Fitridge R . Causes, prevention, and management of diabetes-related foot ulcers. Lancet Diabetes Endocrinol. (2024) 12:472–82. doi: 10.1016/S2213-8587(24)00110-4, PMID: 38824929

[9] Younes NA Albsoul AM Awad H . Diabetic heel ulcers: a major risk factor for lower extremity amputation. Ostomy Wound Manage. (2004) 50:50–60., PMID: 15218204

[10] Saleem S Hayat N Ahmed I Ahmed T Rehan AG . Risk factors associated with poor outcome in diabetic foot ulcer patients. Turk J Med Sci. (2017) 47:826–31. doi: 10.3906/sag-1602-119, PMID: 28618729

[11] Moon KC Kim KB Han SK Jeong SH Dhong ES . Risk factors for major amputation on hindfoot ulcers in hospitalized diabetic patients. Adv Wound Care (New Rochelle). (2019) 8:177–85. doi: 10.1089/wound.2018.0814, PMID: 31737413 PMC6855283

[12] Dong T Huang Q Sun Z . Antibiotic-laden bone cement for diabetic foot infected wounds: A systematic review and meta-analysis. Front Endocrinol (Lausanne). (2023) 14:1134318. doi: 10.3389/fendo.2023.1134318, PMID: 37008902 PMC10060955

[13] Liu C You JX Chen YX Zhu WF Wang Y Lv PP . Effect of induced membrane formation followed by polymethylmethacrylate implantation on diabetic foot ulcer healing when revascularization is not feasible. J Diabetes Res. (2019) 2019:2429136. doi: 10.1155/2019/2429136, PMID: 31828156 PMC6885796

[14] Dai J Zhou Y Mei S Chen H . Application of antibiotic bone cement in the treatment of infected diabetic foot ulcers in type 2 diabetes. BMC Musculoskelet Disord. (2023) 24:135. doi: 10.1186/s12891-023-06244-w, PMID: 36810078 PMC9942328

[15] Guo H Xue Z Mei S Li T Yu H Ning T . Clinical efficacy of antibiotic-loaded bone cement and negative pressure wound therapy in multidrug-resistant organisms diabetic foot ulcers: a retrospective analysis. Front Cell Infect Microbiol. (2024) 14:1521199. doi: 10.3389/fcimb.2024.1521199, PMID: 39831106 PMC11739815

[16] Zhong M Guo J Qahar M Huang G Wu J . Combination therapy of negative pressure wound therapy and antibiotic-loaded bone cement for accelerating diabetic foot ulcer healing: A prospective randomised controlled trial. Int Wound J. (2024) 21:e70089. doi: 10.1111/iwj.70089, PMID: 39379061 PMC11461018

[17] Arora M Chan EK Gupta S Diwan AD . Polymethylmethacrylate bone cements and additives: A review of the literature. World J Orthop. (2013) 4:67–74. doi: 10.5312/wjo.v4.i2.67, PMID: 23610754 PMC3631954

[18] Chang S Jian Y Liu C Dal Prà I Armato U Chen X . Combining antibiotic-loaded bone cement-based free vastus lateralis muscle-sparing flap with split-thickness skin grafts: A reliable strategy for reconstructing diabetic foot ulcers at non-weight-bearing areas. Int Wound J. (2024) 21:e14900. doi: 10.1111/iwj.14900, PMID: 38705731 PMC11070315

[19] Pelissier P Masquelet AC Bareille R Pelissier SM Amedee J . Induced membranes secrete growth factors including vascular and osteoinductive factors and could stimulate bone regeneration. J Orthop Res. (2004) 22:73–9. doi: 10.1016/S0736-0266(03)00165-7, PMID: 14656662

[20] Yang C Wang D . Antibiotic bone cement accelerates diabetic foot wound healing: Elucidating the role of ROCK1 protein expression. Int Wound J. (2024) 21:e14590. doi: 10.1111/iwj.14590, PMID: 38531354 PMC10965272

[21] Chang S Zhang F Chen W Zhou J Nie K Deng C . Outcomes of integrated surgical wound treatment mode based on tibial transverse transport for diabetic foot wound. Front Surg. (2022) 9:1051366. doi: 10.3389/fsurg.2022.1051366, PMID: 36726959 PMC9885215

[22] Mathew G Agha R Albrecht J Goel P Mukherjee I Pai P . STROCSS 2021: Strengthening the reporting of cohort, cross-sectional and case-control studies in surgery. Int J Surg. (2021) 96:106165. doi: 10.1016/j.ijsu.2021.106165, PMID: 34774726

[23] Van Netten JJ Bus SA Apelqvist J Chen P Chuter V Fitridge R . Definitions and criteria for diabetes-related foot disease (IWGDF 2023 update). Diabetes Metab Res Rev. (2024) 40:e3654. doi: 10.1002/dmrr.3654, PMID: 37186781

[24] Ji S Liu X Huang J Bao J Chen Z Han C . Consensus on the application of negative pressure wound therapy of diabetic foot wounds. Burns Trauma. (2021) 9:tkab018. doi: 10.1093/burnst/tkab018, PMID: 34212064 PMC8240517

[25] Chang S Yang W Song H Chen W Zhou J Zhang F . Effectiveness of tibial transverse transport combined with modified neurolysis in treatment of diabetic foot ulcers. Zhongguo Xiu Fu Chong Jian Wai Ke Za Zhi. (2023) 37:1410–7. doi: 10.7507/1002-1892.202306016, PMID: 37987053 PMC10662404

[26] Cai Y Zhang F Feng J Wu B Li H Xiao S . Long-term follow-up and exploration of the mechanism of stromal vascular fraction gel in chronic wounds. Stem Cell Res Ther. (2023) 14:163. doi: 10.1186/s13287-023-03389-2, PMID: 37337292 PMC10280847

[27] Austin PC . Optimal caliper widths for propensity-score matching when estimating differences in means and differences in proportions in observational studies. Pharm Stat. (2011) 10:150–61. doi: 10.1002/pst.433, PMID: 20925139 PMC3120982

[28] Austin PC . An introduction to propensity score methods for reducing the effects of confounding in observational studies. Multivariate Behav Res. (2011) 46:399–424. doi: 10.1080/00273171.2011.568786, PMID: 21818162 PMC3144483

[29] Kallio M Vikatmaa P Kantonen I Lepäntalo M Venermo M Tukiainen E . Strategies for free flap transfer and revascularisation with long-term outcome in the treatment of large diabetic foot lesions. Eur J Vasc Endovasc Surg. (2015) 50:223–30. doi: 10.1016/j.ejvs.2015.04.004, PMID: 26001322

[30] Goudie EB Gendics C Lantis JC 2nd . Multimodal therapy as an algorithm to limb salvage in diabetic patients with large heel ulcers. Int Wound J. (2012) 9:132–8. doi: 10.1111/j.1742-481X.2011.00869.x, PMID: 21951818 PMC7950926

[31] Khoo R Jansen S . Slow to heel: a literature review on the management of diabetic calcaneal ulceration. Int Wound J. (2018) 15:205–11. doi: 10.1111/iwj.12839, PMID: 29431291 PMC7949754

[32] Wang A Lv G Cheng X Ma X Wang W Gui J . Guidelines on multidisciplinary approaches for the prevention and management of diabetic foot disease, (2020 edition). Burns Trauma. (2020) 8:tkaa017. doi: 10.1093/burnst/tkaa017, PMID: 32685563 PMC7336185

[33] Meloni M Izzo V Giurato L Brocco E Gandini R Uccioli L . Limb salvage in diabetic patients with ischemic heel ulcers. Int J Low Extrem Wounds. (2020) 19:275–81. doi: 10.1177/1534734619884438, PMID: 31744357

[34] Ramayanam Navakanth R . Across-sectional observational study on drug utilization and prescribing patterns of antibiotics in lower respiratory tract infections in tertiary care teaching hospital. Asian J Pharmaceut (AJP). (2024) 18:910–3. doi: 10.22377/ajp.v18i3.5642

[35] Onohuean H Olot H Onohuean FE Bukke SPN Akinsuyi OS Kade A . A scoping review of the prevalence of antimicrobial-resistant pathogens and signatures in ready-to-eat street foods in Africa: implications for public health. Front Microbiol. (2025) 16:1525564. doi: 10.3389/fmicb.2025.1525564, PMID: 40270817 PMC12015681

[36] Lyu H Zhu HB Ma YP Zhang YT Hu CT Ying YF . A comparative study of vancomycin loaded bone cement in the treatment of Wagner I-IV diabetic foot. Zhongguo Gu Shang. (2021) 34:947–52. doi: 10.12200/j.issn.1003-0034.2021.10.012, PMID: 34726024

[37] Sun YW Li L Zhang ZH . Antibiotic-loaded bone cement combined with vacuum-assisted closure facilitating wound healing in wagner 3–4 diabetic foot ulcers. Int J Low Extrem Wounds. (2022) 24:15347346221109045. doi: 10.1177/15347346221109045, PMID: 35706401

[38] Crotty KM Yeligar SM . Hyaladherins may be implicated in alcohol-induced susceptibility to bacterial pneumonia. Front Immunol. (2022) 13:865522. doi: 10.3389/fimmu.2022.865522, PMID: 35634317 PMC9133445

[39] Casagrande SS Beccera AZ Rust KF Cowie CC . Opioid prescription and diabetes among Medicare beneficiaries. Diabetes Res Clin Pract. (2023) 196:110240. doi: 10.1016/j.diabres.2023.110240, PMID: 36610545 PMC9974602

[40] Paul P Campbell G Zekeridou A Mauermann M Naddaf E . Diagnosing peripheral neuropathy in patients with alcohol use disorder. Mayo Clin Proc. (2024) 99:1299–305. doi: 10.1016/j.mayocp.2024.02.024, PMID: 39093265

[41] Adam KM Mahmoud SM Mahadi SI Widatalla AH Shawer MA Ahmed ME . (2011). Extended leg infection of diabetic foot ulcers: risk factors and outcome. J Wound Care. 20(9):440–444. doi: 10.12968/jowc.2011.20.9.440, PMID: 22068143

[42] Lu Q Wang J Wei X Wang G Xu Y . (2021). Risk factors for major amputation in diabetic foot ulcer patients. Diabetes Metab Syndr Obes. 14:2019–2027. doi: 10.2147/DMSO.S307815, PMID: 33976562 PMC8106455

[43] Sayyed AA Towfighi P Deldar R Attinger CE Evans KK . (2022). Free flap reconstruction of plantar weight-bearing heel defects: Long-term functional and patient-reported outcomes. Microsurgery. 42(6):538–547. doi: 10.1002/micr.30889, PMID: 35394669

[44] Fitzgerald O’connor EJ Vesely M Holt PJ Jones KG Thompson MM Hinchliffe RJ . A systematic review of free tissue transfer in the management of non-traumatic lower extremity wounds in patients with diabetes. Eur J Vasc Endovasc Surg. (2011) 41:391–9. doi: 10.1016/j.ejvs.2010.11.013, PMID: 21163675

[45] Suh HS Oh TS Lee HS Lee SH Cho YP Park JR . A new approach for reconstruction of diabetic foot wounds using the angiosome and supermicrosurgery concept. Plast Reconstr Surg. (2016) 138:702e–9e. doi: 10.1097/PRS.0000000000002401, PMID: 27673541

[46] Kotha VS Fan KL Schwitzer JA Youn R Black CK Attinger CE . Amputation versus free flap: long-term outcomes of microsurgical limb salvage and risk factors for amputation in the diabetic population. Plast Reconstr Surg. (2021) 147:742–50. doi: 10.1097/PRS.0000000000007644, PMID: 33587553

[47] Suh HP Kedar DJ Lee YH Lee PH Lee SW Hong JP . Use of recanalized vessels for diabetic foot reconstruction: pushing the boundaries of reconstruction in a vasculopathic lower extremity. Plast Reconstr Surg. (2023) 151:485e–94e. doi: 10.1097/PRS.0000000000009935, PMID: 36730343

[48] Ou S Xu C Yang Y Chen Y Li W Lu H . Transverse tibial bone transport enhances distraction osteogenesis and vascularization in the treatment of diabetic foot. Orthop Surg. (2022) 14:2170–9. doi: 10.1111/os.13416, PMID: 35946439 PMC9483085

[49] Tannemaat MR Datema M Van Dijk JG Midha R Malessy MJA . Decompressive surgery for diabetic neuropathy: waiting for incontrovertible proof. Neurosurgery. (2016) 79:783–5. doi: 10.1227/NEU.0000000000001448, PMID: 27861415

