# Correction: Comparative analysis of the therapeutic effect of antibiotic bone cement on Wagner grade 3 or 4 diabetic foot ulcer in heel and non-heel areas: a retrospective cohort study

**DOI:** 10.3389/fendo.2025.1762804

**Published:** 2025-12-17

**Authors:** Yang Jian, Li Li, Wei Chen, Wenyu An, Runxue Guan, Yanji Zhang, Jiarui Wei, Shusen Chang, Jian Zhou, Kaiyu Nie, Chengliang Deng, Zairong Wei

**Affiliations:** 1 Department of Burns and Plastic Surgery, Affiliated Hospital of Zunyi Medical University, Zunyi, Guizhou, China; 2 The 2011 Collaborative Innovation Center of Tissue Damage Repair and Regeneration Medicine, Affiliated Hospital of Zunyi Medical University, Zunyi, Guizhou, China; 3 The Collaborative Innovation Center of Tissue Damage Repair and Regeneration Medicine, Zunyi Medical University, Zunyi, Guizhou, China; 4 Guizhou Biofabrication Laboratory, Affiliated Hospital of Zunyi Medical University, Zunyi, Guizhou, China

**Keywords:** diabetic foot ulcer, antibiotic bone cement, major amputation, free flap, tibial transverse transport

There was a mistake in **Figure 1** as published. The number of patients in the Non-heel group was incorrectly stated as “52”. The correct number is “42”. The corrected **Figure 1** appears below.

**Figure 1:**
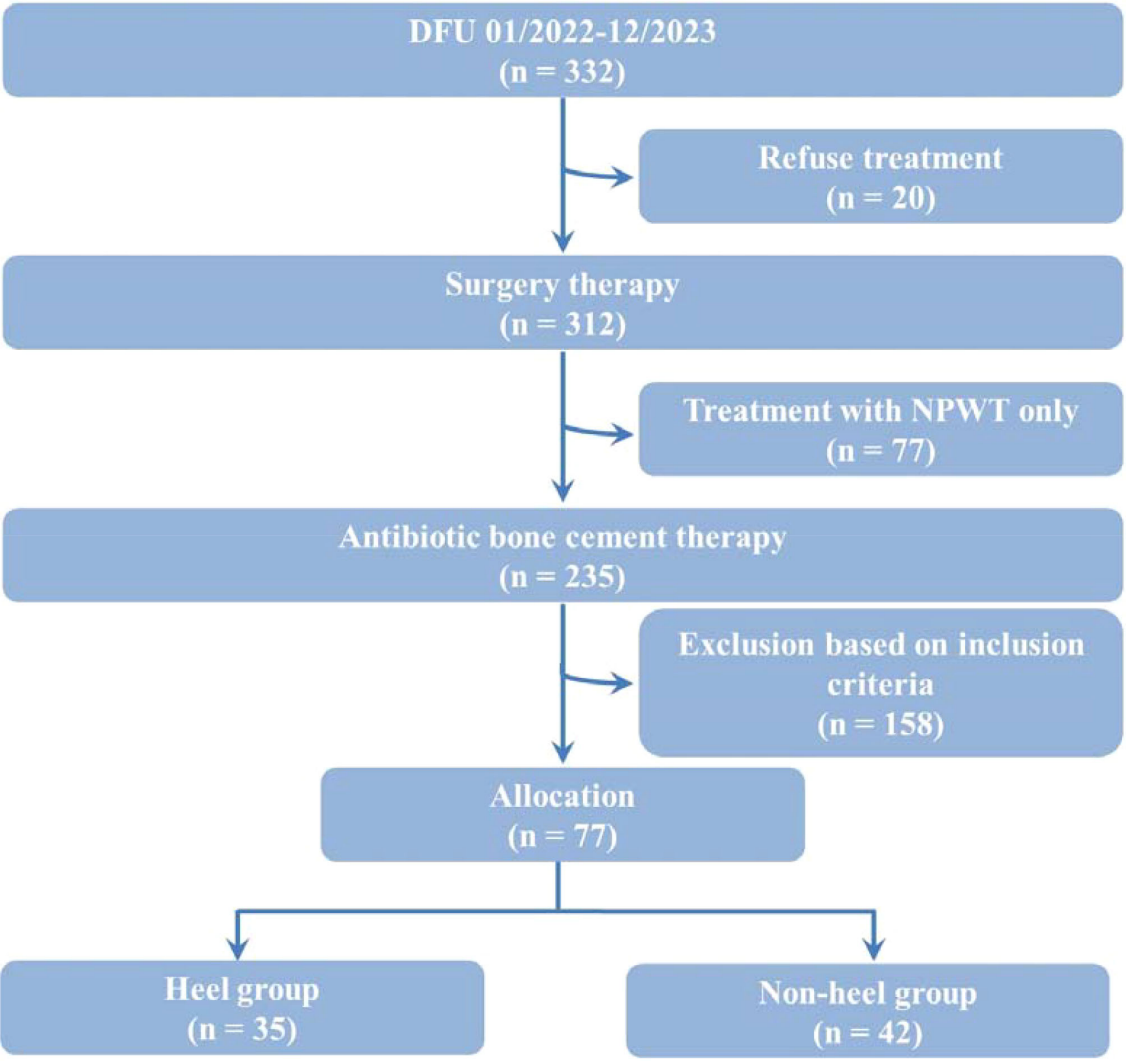
Schematic flow diagram of the study. DFU, diabetic foot ulcer; NPWT, negative pressure wound therapy.

The original version of this article has been updated.

