# Supplementary material for: Comparative analysis of the therapeutic effect of antibiotic bone cement on Wagner grade 3 or 4 diabetic foot ulcer in heel and non-heel areas: a retrospective cohort study
Source: Front Endocrinol (Lausanne). 2025 Nov 26;16:1662731. doi: 10.3389/fendo.2025.1662731 (PMC12689374; doi:10.3389/fendo.2025.1662731)
Supplement: Supplementary file 1 [file Table1.docx]

Supplementary table 1. Clinical characteristics of the enrolled patients after PSM

|  | Heel group (n = 25) | Non-heel group (n = 25) | *P*-value |
| --- | --- | --- | --- |
| Age (years) | 58.08 ± 9.93 | 58.16 ± 11.55 | 0.98 ^a^ |
| Gender, n (%) |  |  | 1.00 ^b^ |
| Male | 17 (68.00) | 18 (72.00) |  |
| Female | 8 (32.00) | 7 (28.00) |  |
| DM duration (years) M (IQR) | 8.00 (3.50, 10.00) | 9.00 (4.50, 10.35) | 0.70 ^c^ |
| DFU duration (months) M (IQR) | 1.00 (0.33, 2.00) | 0.80 (0.50, 2.00) | 0.88 ^c^ |
| ABI | 0.80 ± 0.13 | 0.80 ± 0.14 | 0.96 ^a^ |
| Ulcer size (cm^2^) | 69.00 ± 33.34 | 70.32 ± 29.51 | 0.88 ^a^ |
| Hb (g/L) | 104.96 ± 23.72 | 102.56 ± 21.28 | 0.71 ^a^ |
| Albumin (g/L) | 31.57 ± 5.70 | 31.38 ± 5.24 | 0.90 ^a^ |
| Admission FBG (mmol/L) | 10.98 ± 3.61 | 10.82 ± 5.17 | 0.90 ^a^ |
| HbA1c (%) | 9.00 ± 2.23 | 8.59 ± 1.63 | 0.46 ^a^ |
| First admission WBC (×10^9^/L) | 11.94 ± 3.91 | 10.84 ± 4.32 | 0.35 ^a^ |
| Smoking, n (%) |  |  | 1.00 ^b^ |
| Smoker | 16 (64.00) | 15 (60.00) |  |
| Non-smoker | 9 (36.00) | 10 (40.00) |  |
| Alcohol abuse, n (%) |  |  | 1.00 ^b^ |
| Drinker | 18 (72.00) | 17 (68.00) |  |
| Non-drinker | 7 (28.00) | 8 (32.00) |  |
| Ulcer site, n (%) |  |  |  |
| Left | 16 (64.00) | 14 (56.00) | 0.77 ^b^ |
| Right | 9 (36.00) | 11 (44.00) |  |
| Wagner grade, n (%) |  |  | 1.00 ^b^ |
| 3grade | 11 (44.00) | 12 (48.00) |  |
| 4grade | 14 (56.00) | 13 (52.00) |  |
| Diabetic nephropathy, n (%) | 5 (20.00) | 2 (8.00) | 0.41 ^d^ |
| BC, n (%) |  |  | 0.79 ^e^ |
| Negative | 3 (12.00) | 1 (4.00) |  |
| *Staphylococcus aureus* | 9 (36.00) | 6 (24.00) |  |
| *Pseudomonas aeruginosa* | 2 (8.00) | 2 (8.00) |  |
| *Baumanii* | 3 (12.00) | 2 (8.00) |  |
| *Escherichia coli* | 1 (4.00) | 2 (8.00) |  |
| Mixed infection | 5 (20.00) | 9 (36.00) |  |
| Other | 2 (8.00) | 3 (12.00) |  |

PSM: propensity score matching; DM: diabetes mellitus; DFU: diabetic foot ulcer; ABI: ankle-brachial index; Hb: hemoglobin; FBG: fasting blood glucose; HbA1c: glycosylated hemoglobin A1c; WBC: white blood cell; BC: bacterial culture. ^a^ Student *t*-tests was used for comparison between the two groups; ^b^ Pearson chi-square test was used for comparison between the two groups; ^c^ Mann-Whitney *U* test was used for comparison between the two groups; ^d^ Yates continuity correction was used for comparison between the two groups; ^e^ Fisher's exact test was used for comparison between the two groups.
